# The thermal effects of lavage on 57 ox femoral heads prepared for hip resurfacing arthroplasty

**DOI:** 10.3109/17453674.2013.845812

**Published:** 2013-10

**Authors:** Richard P Baker, Michael R Whitehouse, Angus Maclean, Ashley W Blom, Gordon C Bannister

**Affiliations:** The Avon Orthopaedic Centre, Southmead Hospital, Bristol, UK.

## Abstract

**Background and purpose:**

Previously, we have documented surface temperatures recorded by thermography great enough to cause osteonecrosis of the femoral head during hip resurfacing. We now performed an in vitro investigation with 3 questions: (1) whether water irrigation reduced bone surface temperature, (2) whether external bone temperatures were similar to core temperatures, and (3) whether blunting of the reamer affected temperature generation.

**Methods:**

Using an ox-bone model, 57 femoral heads were peripherally reamed. The surface temperatures of bone were measured using a thermal camera and internal bone temperatures were measured using 2 theromocouples. We measured the effects of cooling with water at room temperature and with ice-cooled water. Progressive blunting of reamers was assessed over the 57 experiments.

**Results:**

Mean and maximum temperatures generated during peripheral reaming were greater when no irrigation was used. Ice-cold saline protected femoral heads from thermal damage. External bone temperatures were much greater than internal temperatures, which were not sufficiently elevated to cause osteonecrosis regardless of lavage. Blunting of the reamer was not found to have a statistically significant effect in this study.

**Interpretation:**

Cooling with ice-cooled water is recommended. Internal bone temperatures are not elevated despite the high surface temperatures reached during femoral head resurfacing.

Hip resurfacing can fail due to osteonecrosis (Fowble et al. 2005, Mont et al. 2007). One of the mechanisms proposed is thermal damage during the procedure (Gill et al. 2007, Baker et al. 2011).

The extent of thermal insult to bone is difficult to quantify. Important factors include the peak temperature and the duration of the thermal insult. With higher temperatures, a shorter exposure is needed to cause injury (Lundskog 1972, Berman et al. 1984). A thermal insult of 47°C for 60 s is the threshold for bone injury (Eriksson and Albrektsson 1983). When bone is exposed to a temperature of 70°C or more, intraoperative macroscopic bone necrosis is seen and cell necrosis occurs within 1s (Moritz and Henriques 1947). There is histological evidence of bone necrosis after exposure to 70°C for 1 min (Berman et al. 1984) and 80°C for 5 s (Lundskog 1972). Thermal insult to bone not only causes cellular damage but alters its mechanical properties, with irreversible changes seen when bone is exposed to temperatures from 50°C to 90°C (Bonfield and Li 1968).

Maximum temperatures of 88°C during reaming of the femoral head have been recorded in vivo and one-third of patients are subjected to temperatures that can induce osteonecrosis. The heat-induced osteonecrosis could damage the interface between the host bone and the implant, and this would affect both cemented and uncemented implants. One study has shown that thermal insult from the exothermic reaction of curing cement is likely during hip resurfacing (Gill et al. 2007), whereas other authors have described lower temperatures in cement mantles from patients undergoing THA (Toksvig-Larsen et al. 1991).

Thermal necrosis could be one factor in the failure of the resurfaced femoral head. Attempts to reduce peak temperature and the duration of exposure may help to reduce any insult to the remaining bone stock in the femoral head. This may be addressed by alteration of surgical technique during femoral head preparation.

We had 3 aims in this study. The first was to establish whether irrigation of the femoral head with chilled saline lowered surface temperatures during reaming. The second aim was to compare the surface and core temperatures of the femoral head using a thermal imaging camera for the surface temperatures and thermocouples deep in the femoral head for the core temperatures. The third aim was to examine the effect of progressive blunting of reamers on surface temperature.

## Materials and methods

A laboratory model was chosen. An in vivo model was discounted as it would have been difficult to achieve the numbers of subjects required to assess progressive blunting of reamers and ensure that the same reamer was used in all cases. Furthermore, measurement of internal and external temperatures concomitantly would have been impractical during the mechanical preparation of the femoral head.

### In vitro model

We used ox femoral heads as our model, as they are similar in shape and size to large human femoral heads and they have an emissivity similar to that of human bone (Baker et al. 2011). Currently, cattle in the UK are slaughtered at a similar age due to current government guidelines following the BSE and CJD scare in the 1990s. The uniform age of slaughter helps standardize the size of the femoral head. Ox bones were obtained from a local meat-processing facility, from cattle that were slaughtered at 2.5 years old.

Peripheral reaming was chosen as the aspect of femoral head preparation to be studied, as in vivo it achieved temperature elevation above the critical levels of 47°C degrees—at which bone necrosis occurs—for the longest time compared to any other stage of the operation (Baker et al. 2011).

The top of the femoral head was removed to form a flat surface. The greater trochanter was resected 10 cm from the flat surface of the caput. A standard Adept guide wire (Finsbury/MatOrtho, Leatherhead, UK) was then passed down the center of the femoral head into the femoral neck.

A flat circular metal jig with a diameter that matched the 48-mm Adept peripheral reamer was passed centrally over the guide wire. The jig has 2-mm pre-drilled channels in it with the center of each channel drilled at a known distance from the central hole. The jig was made with a thickness of 1 cm after trials had shown that thinner jigs afforded less control over K-wire placement and permitted more accurate placement of the thermocouples. Using the jig, 2 channels were made with a 2-mm K-wire into the bone. The first was made 2 mm from the edge of the peripheral cutting tool. The first channel was made to determine temperatures close to the surface of the bone. A second channel was created 10 mm from the center of the circle, to analyze changes in internal bone temperatures.

**Figure 1.**
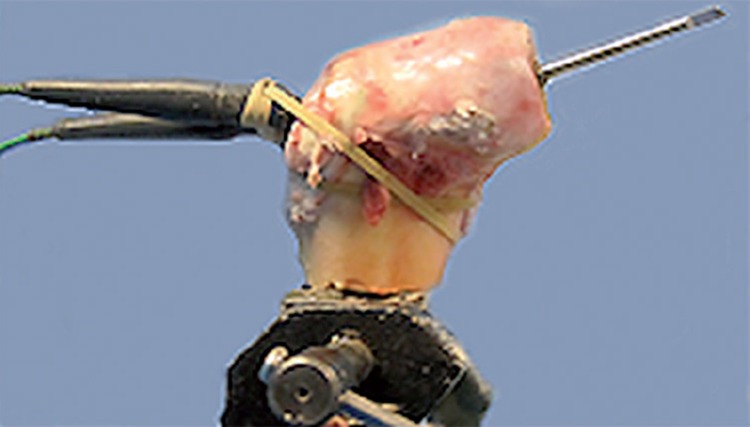
Ox-bone model ready for peripheral reaming and temperature measurement.

The central canal was then made by over-drilling the Adept guide wire. The central peg was then inserted according to standard operative technique. 1 thermocouple was then inserted retrograde up each pre-drilled tunnel, and was kept in place with an elastic band (Figure 1).

### Temperature measurement

External temperatures were measured using a thermal camera (ThermaCam FLIR Systems infra-red A320; Thermascan, Bedford, UK). The camera was mounted 1.2 m from the bovine femoral head. Images were captured at 1 Hz during peripheral reaming. Images were analyzed using ThermaCam Researcher Pro 2.9 software (FLIR Systems). Maximum and mean temperatures (in °C) and duration of reaming (in s) were recorded. Internal bone temperatures were measured using 2 K-type light-duty thermocouples (Cat. no. 342-8883; RS Components Ltd., Corby, UK). The temperatures obtained from the 2 thermocouples were recorded every 250 mS on a PicoLog data recorder (Cat. no. 492-5105; Pico Technologies Ltd., St. Neots, UK).

### Experiment

60 heads were peripherally reamed in succession. The experiments were performed strictly rotating 3 conditions: no lavage, lavage with water at 20°C (range 19–22°C), and lavage with chilled water at 0.8°C (range 0.6–1.2°C). An assistant applied water as a continuous stream from 2 successive 50-mL syringes during reaming. Both emptied over a 10-s period (flow rate: 5 mL/sec).

The above variables were tested in rotation, to control for the effect of sequential blunting of reamers.

### Statistics

The Kolmogorov-Smirnov test was applied to the data to determine whether they had a Gaussian distribution. Inter-group comparison was made using ANOVA between the 3 groups for time of reaming, core temperatures, and surface temperatures. Observations were independent; femoral head samples were unilateral with no bilateral femoral heads included in the study. Subgroup comparisons were made with parametric data using Student’s t-test. Blunting was assessed with Pearson’s correlation coefficient when the data were parametric and Spearman’s rank correlation when they were non-parametric.

## Results

57 experiments were completed. 3 more were planned, but the specimens fractured while being prepared. There were 19 in each group (group 1: no lavage; group 2: water lavage at 20°C; group 3: water lavage at 0.8°C). In 5 experiments, images were not captured by the thermal camera (experiments 3, 9, 10, 12, and 14); in 6 others, the peripheral thermocouple was damaged during reaming and data acquisition failed (experiments 18, 21, 42, 45, 50, and 53) and 1 internal thermocouple reading failed (experiment 42).

### Surface temperatures (thermal camera)

**Figure 2.**
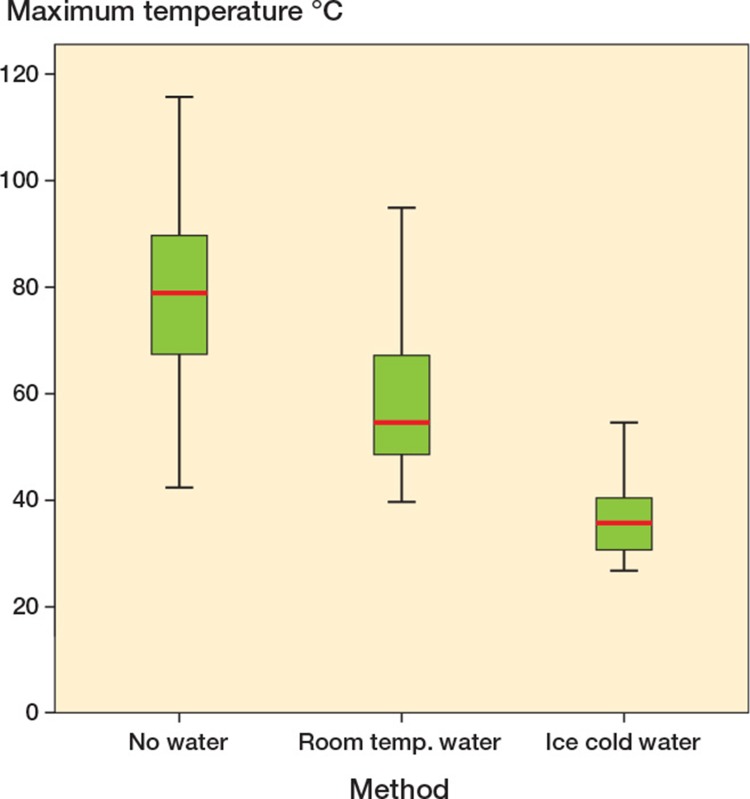
Maximum temperatures recorded from thermal camera.

52 full sets of data were available for analysis (18 in each of groups 1 and 2, and 16 in group 3). The mean maximum temperatures generated were 79°C (SD 18) in group 1, 58°C (SD 15) in group 2, and 37°C (SD 7.0) in group 3 (Figure 2). ANOVA revealed significant differences between the groups (p ≤ 0.001). Both group 2 (p = 0.001) and group 3 (p < 0.001) had significantly lower temperatures than group 1. Group 3 had significantly lower temperatures than group 2 (p < 0.001).

This was reproduced when mean overall temperatures generated were analyzed. Both group 2 (p < 0.001) and group 3 (p < 0.001) showed significantly lower temperatures than group 1. Group 3 had the lowest temperatures of all (p < 0.001). Group 3 had no mean temperatures greater than 30°C; maximum temperatures were low at 37°C (95% CI: 33–40).

No bovine femoral heads in group 3 achieved a temperature above 47°C for 1 min, which is the temperature above which osteocyte death occurs. In group 2, 2 heads were subjected to temperatures of > 80°C for more than 5 s and a further 7 specimens reached temperatures of > 56°C for more than 30 s. In group 1, 9 experiments gave a temperature of > 80°C for more than 5 s and a further 6 gave temperatures of > 56°C for more than 30 s.

### Core temperatures generated (thermocouple)

Core temperatures generated in the bone (Table 1) were much lower than the external temperatures generated. The highest temperatures were recorded in group 1 (no lavage).

**Table 1.** Internal temperatures recorded

Cooling method	Thermocouple	Temperature, °C	
		mean	range
No water	Central	27	20–53
	Peripheral	34	24–57
Water at room	Central	26	21–40
temperature	Peripheral	30	22–38
Ice-cold water	Central	26	18–42
	Peripheral	30	19–48

Both the peripherally placed thermocouples (p = 0.08) and the centrally placed ones (p = 1.0) showed that the temperatures generated were similar between groups.

### Duration of reaming

The mean duration of reaming was 27 (9–47) s in group 1, 29 (10–55) s in group 2, and 31 (8–69) s in group 3. The duration of reaming was similar between the 3 groups (Figure 3) (ANOVA, p = 0.7).

### Effect of blunting

There was no change in the duration of reaming as the experiments progressed. There was also no evidence of increased temperature generation as the experiments progressed (Table 2), with the exception of the peripheral thermocouple, which showed a moderate trend of increased temperatures (correlation coefficient: 0.57).

**Table 2.** Pearson’s correlation coefficient for blunting of reamers

Variable	n	Kolmogorov Smirnov test	Pearson’s correlation coefficient	p-value
Thermal camera, mean	52	0.49	–0.07	0.6
Peripheral thermocouple	51	0.04	0.57 **^a^**	< 0.001
Thermal camera, max	52	0.55	–0.03	0.8
Central thermocouple	56	0.19	0.21	0.1
Amperes	57	0.94	0.05	0.7
Time, s-	52	0.25	0.15	0.3

a Spearman’s rank correlation coefficient was used, as the data were non-parametric.

## Discussion

Use of lavage during reaming of the femoral head resulted in lower maximum and mean surface temperatures. The effect was significantly enhanced by using lavage at 0.8°C. This was a surface phenomenon, and core bone temperatures were unaffected by lavage and remained low during reaming—both with and without lavage.

In all but 3 cases, reaming without lavage resulted in temperatures above the threshold known to be associated with osteocyte injury (56°C). Those that were cooled with water at room temperature had a mean temperature of 58°C, with a maximum recorded temperature of 95°C. Temperatures in this group, despite cooling, were still high enough to lead to cellular injury in 9 of 19 experiments.

We did not observe any effect of blunting and surrogate measures of blunting (time) were redundant. Only in 1 measurement, from the peripheral thermocouple, was an increase in temperature noted as the experiments progressed.

The 48-mm reamer cut into uniform cancellous bone, and we hoped that it would provide a uniform region of bone to measure the effect of blunting. Further work in this area would involve increasing the number experiments and exploring more sensitive measurements of blunting such as the work/force required to continue reaming.

The strength of the ox femoral head model is its availability and similar size to the femoral head of the young human male into whom hip resurfacings are implanted.

Temperatures high enough to induce bone injury during tibial resection in knee arthroplasty have been demonstrated, with lower temperatures recorded at 2-mm and 3-mm depths from the cutting surface (Toksvig-Larsen and Ryd 1989). These findings are similar to our own, showing that heat conduction in bone is poor and that the thermal insult is mainly at the level of the bone-tool interface.

The use of saline to cool bone during surgery has been studied previously (Matthews and Hirsch 1972, Krause et al. 1982). Krause et al. (1982) achieved this with higher flow rates of saline. An internally cooled saw blade has been shown to reduce interface temperatures during knee arthroplasty (Toksvig-Larsen et al. 1990) when irrigation with saline had only a minimal effect (Toksvig-Larsen and Ryd 1989). In contrast, greater temperatures have been observed with saline cooling when drilling into human femoral cortical bone with a 3-mm twist drill at 20,000 rpm (Eriksson et al. 1984). Continuous lavage has also been shown to reduce temperatures associated with cement polymerization during acetabular cementation (Wykman 1992).

Force applied during bone drilling has been shown to be more important than the speed of drilling regarding the maximum temperatures produced (Matthews and Hirsch 1972). Greater force reduced temperatures and reduced the time of bone exposure to temperatures of more than 50°C (Bachus et al. 2000). However, worn drills caused greater temperature changes than sharp drills (Matthews and Hirsch 1972, Natali et al. 1996).

Sharp surgical instruments should reduce thermal damage (Matthews and Hirsch 1972, Natali et al. 1996) as they reduce the length of time and force needed for femoral head preparation.

Caution should be used when extrapolating our data from ox bone to human bone. The temperatures achieved in our experiment were, however, similar to those temperatures seen in vivo for hip resurfacing (Baker et al. 2011). Human bone in vivo was subjected to temperatures great enough to cause cellular injury in one-third of cases. Reducing this risk can only be beneficial to the patient.

It is likely that cooling with saline would be equally effective in human bone. Whether the saline should be ice-cold or at room temperature requires further work in patients undergoing surface replacement arthroplasty. However, ice-cold saline would be unlikely to cause harm to the patient and we feel that it should be advocated for patients currently undergoing surface replacement arthroplasty.

The use of lavage and only using sharp instruments for SRA is a practical step forward to help reduce damage to the femoral head.
